# Predicting Depressive Symptoms From Cross‐Sectional and Longitudinal Data Using XGBoost–SHAP and Cross‐Lagged Model

**DOI:** 10.1155/da/4401725

**Published:** 2026-10-27

**Authors:** Jing Jie, Ming Yin, Jiayue Zeng, Songlin Yang

**Affiliations:** ^1^ State Key Laboratory of Digital Medical Engineering Key Laboratory of Biomedical Engineering of Hainan Province School of Biomedical Engineering Hainan University Sanya Hainan 572024 China hainu.edu.cn; ^2^ School of Biomedical Engineering Sichuan University Chengdu China scu.edu.cn

**Keywords:** COVID-19 pandemic, cross-lagged panel model, depressive symptoms, machine learning, neuroticism, social support

## Abstract

**Objective:**

University students face significant academic, social, and career‐planning challenges. Under such stressors, deficient emotion regulation may increase vulnerability to depressive states, in which persistent symptoms could progress to clinical depression. This study investigates key risk and protective factors for depressive symptoms among university students, including negative life events, neuroticism, and social support. A longitudinal framework was further employed to assess changes in depressive symptoms before and after the COVID‐19 pandemic, with the goal of informing early identification and intervention strategies.

**Methods:**

The dataset included 18 024 valid responses from baseline assessments. Follow‐up assessments conducted 1 year later included 6129 participants. The extreme gradient boosting (XGBoost) model with SHapley Additive exPlanations (SHAP) was used to quantify the relative importance of specific factors (e.g., negative life events and social support) on depressive symptoms, while a cross‐lagged panel model examined longitudinal directional associations between these factors and depressive symptoms.

**Results:**

The prevalence of depressive symptoms was 15.2% among the student sample. Significant correlations were observed between symptom severity and adverse family relationships, childhood trauma, prior suicide attempts, and psychiatric history. Neuroticism and negative life events showed significant positive associations with depressive symptoms, while subjective support and support utilization demonstrated negative associations with symptom severity. Longitudinal analysis revealed an increase in both symptom severity and depression detection rates following the COVID‐19 pandemic. The cross‐lagged panel model identified neuroticism as a significant positive longitudinal predictor of depressive symptoms, whereas subjective support showed a significant negative longitudinal association with subsequent depressive symptoms.

**Conclusion:**

This study identifies neuroticism as an important longitudinal risk indicator for depressive symptoms and subjective support as a protective indicator, while negative life events showed significant cross‐sectional associations with symptom severity. The findings demonstrate how integrating longitudinal designs with interpretable machine learning elucidates complex mental health dynamics in student populations, providing an evidence‐based framework for targeted interventions within campus mental health policies.

## 1. Introduction

The prevalence and impact of depression as a major psychological disorder have been underestimated by the general public. The number of individuals affected by depression is significant, with an estimated 3.8% (~280 million) of the global population experiencing depression. This figure is further supported by studies indicating that over 350 million individuals are affected by depression [1, 2]. Additionally, research suggests that nearly one in five individuals will experience depression by the age of 75, with lifetime prevalence rates reaching as high as 10.6% [3] or above [4]. In particular, since the COVID‐19 pandemic, the number of individuals diagnosed with depression has increased by 53.2 million, representing a growth rate of 27.6%. This increase has been most pronounced among individuals aged 25 years and below [5]. Depression not only affects an individual’s performance at work but may also have a negative impact on social relationships. The 2022 National Blue Book on Depression revealed that depression is highly prevalent among Chinese students, with 50% of those affected being enrolled in school and 41% having withdrawn from their studies due to depression. In intimate relationships, individuals with depression are more likely to experience low rates of marital satisfaction and high rates of marital dissolution [6]. Furthermore, depression is a significant contributor to the global burden of disease [7]. Suicide is also a major public health concern among adolescents and young adults [8].

Depressive symptoms are a general term for a series of symptoms, including a depressed mood, decreased interest, and physical discomfort [9]. If left unaddressed, this negative mood state may develop into depression [10]. It has been proposed that depressive symptoms, minor depression, dysthymia, and major depressive disorder represent a continuum of depressive symptom severity in unipolar major depressive disorder. Each level of this continuum is associated with a significant stepwise increment in psychosocial disability [11]. College students, who are experiencing a multitude of pressures during a crucial period of development, are at an elevated risk for depression [12]. Consequently, a comprehensive examination of the risk and protective factors associated with depressive symptoms among college students is imperative for anticipating the trajectory of depressive symptoms, which can facilitate the reduction of the incidence and severity of depressive symptoms and prevent them from progressing into more severe mental health issues.

The symptoms of depression are the result of a complex psychological state that is formed by the combined effects of multiple dimensions, including physiological, psychological, and socioenvironmental factors. At the physiological level, there is a substantial body of evidence that genetic factors play an important role in the occurrence of depressive symptoms [13]. Epidemiological observations indicate a pronounced familial clustering in major depressive disorder, whereby first‐degree relatives manifest a substantially heightened susceptibility compared to baseline risks observed in the general populace [14, 15]. Early trauma events, such as the loss of family members, sexual abuse, and emotional abuse, are more closely associated with the occurrence of depressive symptoms [16]. Furthermore, personality traits, such as neuroticism, have been identified as significant risk indicators for depressive symptoms [17]. Social support has been established as a protective factor against depression, with the capacity to moderate the relationship between stressful life events and depressive symptoms [17, 18]. An understanding of these factors and their interactions can inform the development of effective prevention strategies and interventions with the aim of reducing the risk of depressive symptoms and improving patients’ mental health.

However, the majority of current research is focused on non‐healthy groups [19] and special groups [20, 21], with inadequate attention paid to depressive symptoms in the general college student population. In terms of predictive methodology, even emerging machine learning‐based depression prediction studies (including those using extreme gradient boosting [XGBoost] and other ensemble learning algorithms widely applied in mental health risk assessment [22, 23]) mostly prioritize model prediction accuracy while lacking in‐depth parsing of the relative importance of multidimensional influencing factors in nonclinical college samples and rarely combining longitudinal tracking designs to examine directional associations between predictors and depressive symptoms over time.

Furthermore, existing studies are often limited to a single dimension of risk factors, lack dynamic longitudinal considerations, and have insufficient exploration of the differential protective mechanisms of social support based on the stress‐buffering theory [24]. Meanwhile, large‐scale causal inference studies are scarce, which limits the comprehensive understanding of the formation mechanism of depressive symptoms and the development of targeted intervention strategies. To address the above gaps, this study used standardized scales to collect cross‐sectional data, to analyze the effects of multiple factors, including social support, neuroticism, and negative life events, and to describe the epidemiological characteristics of depressive symptoms in college students. Meanwhile, a 1‐year follow‐up study was conducted to explore dynamic changes in depressive symptoms during the pandemic and to examine longitudinal directional associations between depressive symptoms and their influencing factors.

## 2. Materials and Methods

### 2.1. Participants

This study conducted an online questionnaire survey at a key comprehensive university under the jurisdiction of Hainan Province, located in South China. The respondents were from various regions of the Chinese Mainland, with a wide distribution and favorable regional representativeness of the sample. All participants were volunteers and provided informed consent prior to the administration of the survey. The participants were at least 18 years of age, demonstrated an ability to comprehend the content of the questionnaire, and exhibited the capacity to complete the questionnaire on an electronic device.

A total of 19 561 valid questionnaires were initially obtained from three undergraduate grades (excluding the graduating cohort), and 1537 responses were discarded due to insufficient quality, as evidenced by excessive errors on the attention check items. Consequently, 18 024 valid questionnaires were available for analysis, of which 8511 were completed by male respondents and 9513 by female respondents (19.17 ± 1.20 years). Due to graduation and internships, some participants from the two grade levels were unable to complete the retest. Therefore, only the youngest undergraduate cohort was included in the 1‐year follow‐up survey, with a total of 6129 valid participants (3038 males and 3091 females; mean age = 19.05 ± 0.83 years). To assess potential nonresponse bias, baseline sex, age, and depression scores were compared between follow‐up completers and nonrespondents (Supporting Information 3: Appendix Table S3). Although statistically significant differences were observed in sex, age, and baseline depressive symptom scores between follow‐up completers and nonrespondents, the differences in age and depressive symptom scores were relatively small. These findings suggest that some degree of nonresponse bias cannot be excluded and should be considered when interpreting the longitudinal results.

### 2.2. Measures

#### 2.2.1. The Social Support Rating Scale

The Social Support Rating Scale [25], modified to exclude unsuitable questions, was employed to assess the social support networks of college students. Responses were provided on a four‐point Likert scale. Some sample items include: “How many close friends do you have that you can count on for support and help? (1. None; 2. One or two; 3. Three to five; 4. Six or more),” “Interactions with your classmates. (1. Never care about each other, just nodding acquaintances; 2. May care a little if you are in trouble; 3. Some classmates care about you; 4. Majority of classmates care about you),” and “The support and care you receive from your parents. (1. None; 2. Very little; 3. Moderate; 4. A lot).” The pretest Cronbach’s *α* coefficient was 0.775. Similarly, the posttest *α* coefficient was 0.786.

#### 2.2.2. The Beck Depression Inventory‐II (BDI‐II)

The BDI‐II [26, 27] was employed to assess the presence and severity of depressive symptoms. The scale consists of 21 items, each rated on a four‐point scale from 0 to 3, yielding a total score ranging from 0 to 63, with higher scores indicating greater depressive symptom severity. The pretest and posttest Cronbach’s *α* coefficients were 0.847 and 0.880, respectively.

#### 2.2.3. Adolescent Self‐Rating Life Events Checklist (ASLEC)

The Adolescent Self‐Rating Life Events Checklist (ASLEC) [28] is a tool designed to assess the number of recent negative life events, the total amount of recent stress, and the sensitivity to recent stress in the past 12 months. The ASLEC comprises 27 negative life‐event items. In the present study, five factors, including interpersonal relationships, academic stress, punishment, loss, and health and adaptation, were included in the analyses. The interpersonal relationship dimension reflects negative life events involving difficulties or adverse experiences in interpersonal interactions. Representative items include “Being misunderstood or wrongly blamed by others” and “Having disputes with classmates or close friends.” The scale employs a six‐level scoring method, with 0 indicating that the event has not occurred. In the event of occurrence, the impact on the individual is divided into five levels: 1 = no impact, 2 = mild, 3 = moderate, 4 = severe, and 5 = extremely severe. The pretest Cronbach’s *α* coefficient was 0.931. Similarly, the posttest *α* coefficient was 0.936. The Cronbach’s *α* coefficients for various dimensions of events in this scale are presented in Supporting Information 2: Appendix Table S2.

#### 2.2.4. The Personal Growth Experience Questionnaire

The Personal Growth Experience questionnaire is designed to collect personal information, including educational background, career development, personal achievements, life experience, and other pertinent data. This information is then analyzed and interpreted in order to gain insight into the personal growth process and experience of the individual in question. In addition, the data provides an understanding of the key events encountered as well as how the individual responds to challenges and seizes opportunities.

#### 2.2.5. Eysenck Personality Questionnaire‐Revised Short Form (EPQ‐R‐S)—Neuroticism Subscale

This study assessed participants’ neuroticism using the Neuroticism subscale of the EPQ‐R‐S [29], a widely used self‐report measure for the three core dimensions of the Eysenck personality model. The neuroticism subscale of the EPQ‐R‐S operationalizes individual variance in emotional instability. Specifically, elevated scores typically manifest as a predisposition toward chronic anxiety and affective lability; conversely, lower deciles are characterized by robust emotional equilibrium, evidenced by attenuated affective reactivity and an accelerated physiological return to baseline following emotional arousal. Sample items of the EPQ‐R‐S Neuroticism subscale include “Do you experience fluctuations in your mood?” and “Do you tend to worry?” The subscale demonstrated good internal consistency in the present study, with a Cronbach’s *α* of 0.841 at the pretest and 0.848 at the posttest.

### 2.3. Statistical Analyses

PyCharm 2023.3.1 (Python 3.10.11) and IBM SPSS Statistics 26.0 were employed to carry out a cross‐sectional analysis of college students’ depressive symptoms. This included data processing, descriptive statistical analysis, difference analysis (e.g., chi‐square test), and Pearson correlation analysis. Additionally, Mplus Editor software was utilized to construct a cross‐lagged model for longitudinal analysis.

In this study, five adolescent life‐event factors, two social‐support factors, and neuroticism were included as predictor variables, with the depressive symptom score as the outcome variable. A multiple linear regression model and XGBoost model (version 3.2.0) combined with SHapley Additive exPlanations (SHAP; version 0.49.1) were used to examine associations and quantify the relative importance of these predictors. The cross‐lagged panel model was used to examine longitudinal directional associations between depressive symptoms and predictor variables.

#### 2.3.1. The Multiple Linear Regression Model

Multiple linear regression was used to examine the independent associations between the predictor variables and depressive symptom scores while adjusting for the other variables included in the model [30, 31]. Model performance was evaluated using the coefficient of determination (*R* ^2^), adjusted *R* ^2^, and the overall *F*‐test. Multicollinearity was assessed using the variance inflation factor (VIF), and residual independence was evaluated using the Durbin–Watson statistic. The residual distribution was examined using a histogram, a *Q*–*Q* plot, and the Shapiro–Wilk test [32]. The primary regression model was fitted using the original predictor scales, with both unstandardized coefficients (*B*) and standardized coefficients (*β*) being reported. To assess the robustness of the findings to deviations from residual normality, a sensitivity analysis was additionally conducted using Huber *M*‐estimation robust regression. For direct coefficient comparison between the ordinary least squares (OLS) and robust regression models in the sensitivity analysis, all predictor variables were standardized before model fitting.

#### 2.3.2. The XGBoost–SHAP Model

The XGBoost [33] model was introduced as a means of supplementing and comparing the multiple linear regression model. XGBoost is based on gradient‐boosting decision trees (GBDTs), which improves model predictive performance by iteratively building multiple decision trees to minimize prediction residuals. The objective function of the model was set to reg:squarederror (squared error) to minimize the square of the difference between the predicted and actual values of depressive symptom scores.

On the independent holdout test set, the model achieved *R* ^2^ = 0.5983 and adjusted *R* ^2^ = 0.5974. To interpret the underlying logic of the XGBoost‐predicted depressive symptom scores, we utilized the SHAP framework [34] to isolate the marginal contribution of each constituent feature. Specifically, TreeExplainer, configured with interventional feature perturbation, was implemented to derive SHAP values for the test cohort. The hierarchy of global feature importance was subsequently established via mean absolute SHAP values, thereby quantifying the average impact magnitude of individual predictors on the final model estimations.

The XGBoost hyperparameter configuration was specified with consideration of learning dynamics, model complexity, regularization, and feature subsampling, consistent with the methodological literature on the roles and tunability of key XGBoost hyperparameters [33, 35]. The final configuration used a learning rate of 0.05 with 300 boosting iterations, a maximum tree depth of 5, an L1 regularization parameter of 20, and a column subsampling ratio of 0.35. Specifically, the learning rate controlled the contribution of each boosting step, the number of boosting iterations determined the number of trees, the maximum tree depth constrained tree complexity, L1 regularization penalized leaf weights, and column subsampling introduced feature‐level stochasticity during tree construction. The fitted model was subsequently used for SHAP analysis.

Initial screening of the complete sample (*N* = 18 024) revealed no missing observations, which rendered participant exclusion unnecessary during the preprocessing stage. The independent variables, including measures of interpersonal relationships, academic stress, punishment, loss, health and adaptation, subjective and utilized support, and neuroticism, were obtained as continuous data through questionnaire assessments. Because the feature set consisted exclusively of continuous variables, the model did not require standard encoding techniques like one‐hot or label encoding. This omission inherently reduced potential biases during the subsequent interpretation of SHAP values. For model development, the dataset was divided into training and test sets using an 80/20 ratio. To ensure strict analytical reproducibility, a fixed seed (random state = 42) was applied to all stochastic operations, covering data splitting, model initialization, and the fitting process.

#### 2.3.3. The Cross‐Lagged Model

As the multiple linear regression model and the XGBoost–SHAP model are based on the analysis of cross‐sectional data, it is statistically challenging to ascertain the direction of the relationship between the predictor variables and depressive symptoms. Consequently, following the preliminary screening of predictor variables for depressive symptoms using the aforementioned two models, further longitudinal data was collected and cross‐lagged analysis [36] was employed to examine the longitudinal directional associations between the predictor variables and depressive symptoms. Cross‐lagged analysis represents an analytical strategy used to examine interrelationships or directional associations between variables over time. A cross‐lagged model was constructed using the Mplus Editor software to evaluate the influence of factors at two different time points (the initial data collection was recorded as T1 and the subsequent data acquisition was recorded as T2). The specific construction process of the model is as follows: first, the influencing factors of depressive symptoms and the severity of depressive symptoms in the T1 and T2 periods were analyzed by autocorrelation analysis. This was done in order to investigate the stability and continuity of the variables in the time series, which was helpful to understand the changing trend of each variable at different time points. Subsequently, a cross‐lagged path was established from the influencing factors of depressive symptoms at T1 to the severity of depressive symptoms at T2. This was done to examine whether specific influencing factors at T1 were longitudinally associated with depressive symptoms at T2, thereby clarifying the temporal relationship between these factors and depressive symptom severity.

## 3. Results

### 3.1. Comparison of Depressive Symptoms Among Students With Different Demographic Characteristics

According to the Beck Depression Inventory (BDI) scores, the detection rate of depressive symptoms among college students was 15.2%. Specifically, mild depressive symptoms (scores 14–19) were observed in 9.7% (1749 students), moderate depressive symptoms (scores 20–28) in 4.6% (831 students), and severe depressive symptoms (scores 29–63) in 0.9% (154 students).

Students from financially stable families had significantly lower average depression scores and detection rates compared to those from impoverished families (*χ* ^2^ = 177.74, *p* < 0.001). Compared with individuals who endured traumatic or distressing childhood experiences, students with favorable early‐life backgrounds demonstrated significantly lower depression scores and detection rates (*χ* ^2^ = 1045.62, *p* < 0.001). Students with harmonious parental relationship (*χ* ^2^ = 481.18, *p* < 0.001) had significantly lower average depression scores and detection rates than those from divorced (*χ* ^2^ = 34.78, *p* < 0.001) or single‐parent families (*χ* ^2^ = 33.20, *p* < 0.001). Parental relationship quality was significantly associated with depressive symptoms among the surveyed students. Specifically, those maintaining favorable rapport with their fathers demonstrated lower depression scores and detection rates in comparison to peers subjected to paternal harshness or emotional rejection (*χ* ^2^ = 680.12, *p*<0.001). A corresponding trend was identified regarding maternal influence, where positive engagement was associated with diminished depressive indicators compared to instances of neglect or punishment (*χ* ^2^ = 684.43, *p*<0.001). Notably, the statistical impact of maternal factors on depressive symptoms appeared to be more pronounced than that of paternal variables. Furthermore, psychiatric histories were significantly associated with higher depressive symptom scores and detection rates. This upward trend in both mean scores and detection frequencies was consistent across students with a family history of mental illness (*χ* ^2^ = 221.31, *p* < 0.001) or suicide (*χ* ^2^ = 70.79, *p* < 0.001), as well as those with personal histories of mental disorders (*χ* ^2^ = 731.17, *p* < 0.001) or suicidal behavior (*χ* ^2^ = 501.18, *p* < 0.001). Comprehensive distributions of these variables across different severity levels are documented in Supporting Information 1: Appendix Table S1.

### 3.2. Factors Influencing Depressive Symptoms

Correlation tests were conducted on five factors pertaining to adolescent life events and two factors related to social support, neuroticism, and depression symptom scores. As illustrated in Table 1 and Figure 1, depressive symptoms were found to be positively correlated with interpersonal relationships, academic stress, punishment, loss, health and adaptation, and neuroticism. Among the factors, neuroticism demonstrated the strongest correlation (*r* = 0.61), followed by academic stress and interpersonal relationships. Depressive symptoms exhibited a negative correlation with subjective support and support utilization.

**Figure 1:**
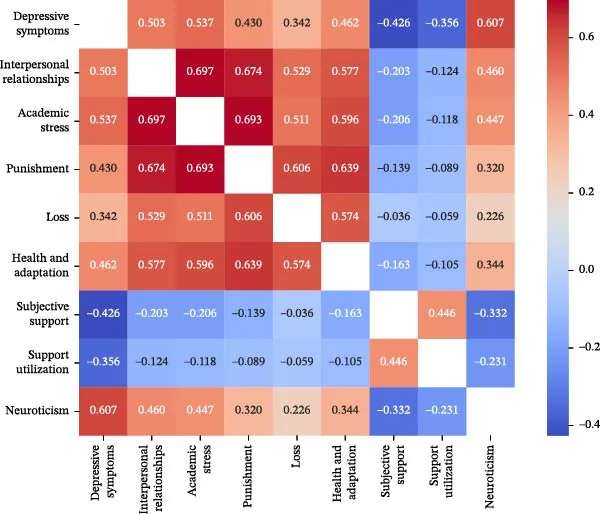
Heatmap of the correlation between influencing factors and depressive symptoms.

**Table 1:** Analysis of the correlation between influencing factors and depressive symptoms.

	Depressive symptoms	Interpersonal relationships	Academic stress	Punishment	Loss	Health and adaptation	Subjective support	Support utilization	Neuroticism
Depressive symptoms	1	—	—	—	—	—	—	—	—
Interpersonal relationships	0.503^∗∗∗^	1	—	—	—	—	—	—	—
Academic stress	0.537^∗∗∗^	0.697^∗∗∗^	1	—	—	—	—	—	—
Punishment	0.430^∗∗∗^	0.674^∗∗∗^	0.693^∗∗∗^	1	—	—	—	—	—
Loss	0.342^∗∗∗^	0.529^∗∗∗^	0.511^∗∗∗^	0.606^∗∗∗^	1	—	—	—	—
Health and adaptation	0.462^∗∗∗^	0.577^∗∗∗^	0.596^∗∗∗^	0.639^∗∗∗^	0.574^∗∗∗^	1	—	—	—
Subjective support	−0.426^∗∗∗^	−0.203^∗∗∗^	−0.206^∗∗∗^	−0.139^∗∗∗^	−0.036^∗∗∗^	−0.163^∗∗∗^	1	—	—
Support utilization	−0.356^∗∗∗^	−0.124^∗∗∗^	−0.118^∗∗∗^	−0.089^∗∗∗^	−0.059^∗∗∗^	−0.105^∗∗∗^	0.446^∗∗∗^	1	—
Neuroticism	0.607^∗∗∗^	0.460^∗∗∗^	0.447^∗∗∗^	0.320^∗∗∗^	0.226^∗∗∗^	0.344^∗∗∗^	−0.332^∗∗∗^	−0.231^∗∗∗^	1

*Note:*  ^∗∗∗^ *p* < 0.001.

A multiple linear regression model was constructed with eight predictor variables: five factors of adolescent life events (interpersonal relationships, academic stress, punishment, loss, and health and adaptation), two factors of social support (subjective support and support utilization), and neuroticism. Depressive symptom scores were used as the outcome variable. The results of the multiple linear regression analyses are presented in Table 2. The model explained 54.4% of the variance in depressive symptom scores (*R^2^* = 0.544 and adjusted *R^2^* = 0.544) and was statistically significant overall (*F* (8, 18 015) = 2684, *p* < 0.001). With the exception of punishment (*p* = 0.553), all other predictors were significantly associated with depressive symptom scores (all *p* < 0.001). The VIF values ranged from 1.263 to 2.680, indicating no evidence of problematic multicollinearity. The Durbin–Watson statistic was 1.983, suggesting no substantial residual autocorrelation. Although punishment was positively correlated with depressive symptoms at the bivariate level (*r* = 0.430, *p* < 0.001), it was not independently associated with depressive symptoms after adjustment for the other predictors. Punishment was also correlated with interpersonal relationships (*r* = 0.674) and academic stress (*r* = 0.693), suggesting that part of its association with depressive symptoms may overlap with that of other life‐event dimensions. Standardized coefficients (*β*) were additionally examined to compare the relative strength of associations across predictors. Neuroticism showed the largest positive standardized coefficient (*β* = 0.350), whereas subjective support (*β* = −0.168) and support utilization (*β* = −0.155) showed negative associations with depressive symptom scores.

**Table 2:** Parameters of multiple linear regression model.

Variables	Unstandardized coefficient		Standardized coefficient *β*	*t*	*p*	Covariance statistics	
	*B*	Standard errors				Tolerances	VIF
Constant	7.069	0.210	—	33.645	<0.001	—	—
Neuroticism	0.693	0.012	0.350	57.792	<0.001	0.691	1.446
Interpersonal relationships	0.098	0.013	0.062	7.809	<0.001	0.403	2.484
Academic stress	0.316	0.014	0.185	22.975	<0.001	0.391	2.561
Punishment	0.005	0.009	0.005	0.593	0.553	0.373	2.680
Loss	0.094	0.013	0.047	7.001	<0.001	0.553	1.809
Health and adaptation	0.291	0.017	0.121	16.894	<0.001	0.492	2.034
Subjective support	−0.397	0.014	−0.168	−28.600	<0.001	0.732	1.365
Support utilization	−0.574	0.021	−0.155	−27.343	<0.001	0.792	1.263

Inspection of the residual histogram showed no prominent multimodality (Supporting Information 5: Appendix Figure S1), whereas the *Q*–*Q* plot indicated deviations from normality, particularly at the distributional extremes (Supporting Information 6: Appendix Figure S2). The Shapiro–Wilk test was also statistically significant (*p* < 0.001), indicating a departure from residual normality. Given the large sample size, the Shapiro–Wilk result was interpreted in conjunction with the graphical diagnostics. A Huber *M*‐estimation robust regression was therefore conducted as a sensitivity analysis to assess the robustness of the regression findings [32].

The Huber *M*‐estimation robust regression yielded a pattern of results that was highly consistent with the OLS model. The seven predictors that were statistically significant in the OLS model remained statistically significant and showed the same direction of association in the robust regression model, whereas punishment remained non‐significant in both models (OLS: *p* = 0.553; robust regression: *p* = 0.405). These findings indicate that the main regression results were robust to the estimation method.

Given the potential for nonlinear relationships and complex interactions among predictors, the XGBoost–SHAP framework was implemented to complement the multiple linear regression analysis. Based on global impact quantified by mean absolute SHAP values, the feature importance ranking identified neuroticism (1.681) as the most influential predictor, followed by academic stress (0.957), subjective support (0.908), and support utilization (0.823). Additional variables contributing to the predictive output included health and adaptation (0.680), interpersonal relationships (0.449), loss (0.265), and punishment (0.166), as detailed in Table 3 and Figure 2. The SHAP summary plot indicated that higher values of most predictors were generally associated with positive contributions to predicted depressive symptom scores, whereas higher subjective support and support utilization tended to contribute negatively to the model predictions.

**Figure 2:**
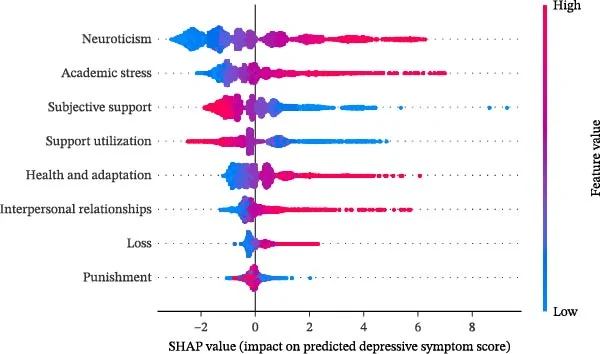
SHAP beeswarm summary plot for the XGBoost regression model predicting depressive symptom scores. Features on the *y*‐axis are ranked in descending order of global predictive importance (quantified by mean absolute SHAP value across test samples). The *x*‐axis represents the SHAP value, which reflects the magnitude and direction of a feature’s impact on predicted depressive symptom scores (positive values = increased predicted scores; negative values = decreased scores). Dot color indicates feature value (red = high; blue = low), with each dot representing a participant from the independent 20% holdout test set.

**Table 3:** Mean absolute SHAP value of characteristic variables.

Variables	Mean absolute SHAP value
Interpersonal relationships	0.449
Academic stress	0.957
Punishment	0.166
Loss	0.265
Health and adaptation	0.680
Subjective support	0.908
Support utilization	0.823
Neuroticism	1.681

### 3.3. Longitudinal Analysis of Depressive Symptom Trajectories

The mean score and detection rate of depressive symptoms were found to be higher in the T2 period than in the T1 period. In particular, the mean score for depressive symptoms in T1 was 7.26 ± 6.60, with a corresponding depressive symptom detection rate of 16.22%. In the second period (T2), the mean score for depressive symptoms was 8.21 ± 7.79, with a detection rate of 21.67%. A total of 4312 individuals exhibited no depressive symptoms in both the T1 and T2 periods. A total of 505 individuals exhibited depressive symptoms in both periods. A total of 823 individuals exhibited no depressive symptoms in T1 but subsequently displayed depressive symptoms in T2. Furthermore, 489 individuals exhibited depressive symptoms in T1 but not in T2. Changes in depressive symptom status between T1 and T2 were assessed using McNemar’s test, which indicated a statistically significant difference (*p* < 0.001).

As illustrated in Table 4, the group without depressive symptoms exhibited lower mean scores for neuroticism, interpersonal relationships, academic stress, and punishment, and higher mean scores for subjective support and support utilization in both the T1 and T2 periods in comparison to the group with depressive symptoms. A differential analysis of the scores for factors affecting depressive symptoms at T1 and T2 was conducted using the Wilcoxon signed‐rank test, with significant results (*p* < 0.001).

**Table 4:** Analysis of influencing factors of depressive symptoms in periods T1 and T2.

Variables	T1		T2	
	No depressive symptoms	Have depressive symptoms	No depressive symptoms	Have depressive symptoms
Interpersonal relationships	8.68 ± 3.96	11.80 ± 4.17	8.44 ± 4.11	11.43 ± 4.13
Academic stress	8.56 ± 3.52	11.52 ± 3.82	8.38 ± 3.80	11.25 ± 4.08
Punishment	7.59 ± 5.84	11.11 ± 6.77	7.12 ± 6.10	10.95 ± 6.93
Loss	4.15 ± 3.58	5.53 ± 3.94	3.96 ± 3.52	5.56 ± 3.77
Health and adaptation	4.02 ± 2.77	5.80 ± 3.22	4.41 ± 2.84	6.39 ± 3.13
Subjective support	14.08 ± 2.42	12.41 ± 2.84	13.09 ± 2.83	11.23 ± 3.01
Support utilization	5.29 ± 1.68	4.33 ± 1.80	5.20 ± 1.82	4.29 ± 1.93
Neuroticism	3.96 ± 2.93	7.87 ± 3.06	3.98 ± 3.08	7.73 ± 3.21

As shown in Table 5, participants who transitioned from no depressive symptoms at T1 to depressive symptoms at T2 generally showed increases in negative life event and neuroticism scores, accompanied by decreases in social support scores. In contrast, participants who transitioned from depressive symptoms at T1 to no depressive symptoms at T2 generally showed decreases in negative life event and neuroticism scores. The largest increase was observed for neuroticism, which increased from 5.28 ± 3.11 to 7.29 ± 3.28. Conversely, the largest decrease was observed for punishment, which decreased from 10.67 ± 6.81 to 7.74 ± 6.27.

**Table 5:** Analysis of factors influencing changes in depressive symptoms.

Variables	No depressive symptoms → Have depressive symptoms		Have depressive symptoms → No depressive symptoms	
Interpersonal relationships	9.91 ± 3.96	11.11 ± 4.03	11.30 ± 4.31	9.35 ± 4.36
Academic stress	9.46 ± 3.67	11.08 ± 4.14	11.20 ± 3.70	9.07 ± 3.94
Punishment	9.16 ± 6.25	10.77 ± 6.94	10.67 ± 6.81	7.74 ± 6.27
Loss	4.79 ± 3.78	5.42 ± 3.77	5.37 ± 4.02	4.45 ± 3.81
Health and adaptation	4.69 ± 2.90	6.26 ± 3.10	5.53 ± 3.19	4.87 ± 2.98
Subjective support	13.57 ± 2.64	11.38 ± 3.03	12.55 ± 2.94	12.32 ± 3.01
Support utilization	5.05 ± 1.73	4.43 ± 1.91	4.52 ± 1.83	4.74 ± 1.82
Neuroticism	5.28 ± 3.11	7.29 ± 3.28	7.54 ± 3.23	5.36 ± 3.30

The cross‐lagged model demonstrated a good fit to the data, with an RMSEA of 0.039 (90% CI [0.036, 0.042]), a comparative fit index (CFI) of 0.980, a TLI of 0.963, and an SRMR of 0.067. The chi‐square to degrees of freedom ratio is 10.39, which exceeds the conventional critical value of 3 or 5. Nevertheless, in the context of large samples, the chi‐square test may be unduly sensitive. It is possible for the chi‐square value to be significant even when the model fit is deemed acceptable [37, 38]. In light of the aforementioned fit indicators, the model demonstrates a satisfactory fit and meets the requisite model evaluation criteria. To enhance the rigor, standardization, and comprehensiveness of our longitudinal analysis, we examined three levels (configural, metric, and scalar) of longitudinal measurement invariance via multigroup CFA prior to the CLPM. Given our large sample, we adopted ΔCFI ≥ −0.01 as the criterion to address the oversensitivity of the chi‐square test. Most scales attained full scalar invariance; the growth scale achieved supported metric invariance (ΔCFI = −0.0034) and partial scalar invariance, which is well justified in large‐sample longitudinal studies [39], ensuring the reliability and validity of subsequent CLPM findings. Detailed results of the longitudinal measurement invariance tests are provided in Supporting Information 4: Appendix Table S4.

As illustrated in Table 6, subjective support showed a significant negative cross‐lagged association with subsequent depressive symptoms (*β* = −0.082, *p* = 0.024), whereas neuroticism showed the strongest positive cross‐lagged association (*β* = 0.236, *p* < 0.001). To further address the suggestion regarding the potential buffering role of social support, we additionally performed a longitudinal moderation analysis among 5410 participants with complete data at both T1 and T2. Using hierarchical regression while controlling for T1 depressive symptoms and gender, results showed that T1 neuroticism significantly positively predicted T2 depressive symptoms (*B* = 0.997, *p* < 0.001), and T1 social support significantly negatively predicted T2 depressive symptoms (*B* = −0.451, *p* < 0.001). However, the interaction term between neuroticism and social support was not significant (*B* = −0.053, *p* = 0.549), suggesting that social support did not significantly buffer the longitudinal impact of neuroticism on depressive symptoms in the present sample.

**Table 6:** Cross‐lagged results of depressive symptoms and influencing factors.

Items	Estimate	S.E.	Est./S.E.	*p*
Interpersonal relationships	−0.033	0.028	−1.206	0.228
Academic stress	−0.001	0.032	−0.034	0.973
Punishment	0.020	0.020	0.985	0.324
Loss	0.014	0.027	0.512	0.609
Health and adaptation	−0.013	0.036	−0.351	0.726
Subjective support	−0.082	0.036	−2.265	0.024
Support utilization	−0.088	0.051	−1.729	0.084
Neuroticism	0.236	0.033	7.213	<0.001

## 4. Discussion

Utilizing both cross‐sectional and longitudinal data, this study explores the intricate determinants and underlying mechanisms that characterize depressive symptoms among college students. The cross‐sectional analysis identifies several high‐risk indicators, including family financial strain, adverse childhood experiences, and harsh parenting, with maternal influence being particularly pronounced. Notably, a personal psychiatric history was also significantly associated with depressive symptoms. This evidence is further extended by longitudinal tracking, which underscores a marked escalation in symptom severity following the pandemic. The longitudinal findings further identified neuroticism as a positive predictor of depressive symptoms and subjective support as a negative predictor, while no significant buffering interaction between neuroticism and social support was observed. Through the synthesis of these environmental, dispositional, and social dimensions, the study presents a refined framework that is crucial for customizing targeted psychological interventions within academic contexts.

### 4.1. Cross‐Sectional Analysis of Depressive Symptoms Among College Students

The analysis of demographic disparities emphasizes a complex interaction of socioeconomic and relational factors influencing depressive outcomes. Specifically, the findings indicate that family economic status, childhood trauma, household cohesion, and psychiatric family history are significantly associated with depressive symptom severity. The markedly lower depressive symptom scores observed among students from harmonious households relative to those in high‐conflict environments lend empirical weight to the family systems theory [40] and reinforce the documented protective capacity of robust domestic support [41]. These findings suggest that a stable and supportive family environment may be associated with greater psychological resilience among college students. Regarding disciplinary dynamics, punitive parenting was associated with poorer psychological outcomes, with particularly marked differences observed across maternal parenting categories. This asymmetry echoes existing longitudinal evidence [42–44], suggesting that the generally greater emotional closeness and daily involvement typical of maternal caregiving may strengthen the effect of maternal parenting style on a child’s psychological development.

Furthermore, the association between financial hardship, adverse childhood events, and symptom manifestation grounds our findings in the psychological stress theory [45]. These stressors likely catalyze maladaptive stress responses, thereby lowering the threshold for depressive onset. A pivotal observation in this study is the disproportionate detection rate of symptoms among individuals with personal or familial histories of psychiatric crisis or suicidal ideation. Notably, personal psychiatric history was strongly associated with current depressive symptoms, alongside significant associations observed for family psychiatric history. While this corroborates the role of biological predispositions [46], the relative dominance of individual clinical history over familial traits presents a nuanced distinction seldom clarified in the extant literature. This divergence necessitates a more granular exploration of the discrete mechanisms through which individual‐level psychiatric trajectories and genetic vulnerabilities independently or synergistically modulate depressive pathology.

### 4.2. Multidimensional Influencing Factors of Depressive Symptoms

By examining the convergence of dispositional traits and external stressors, our investigation elucidates the discrete pathways through which negative life events, neuroticism, and social support shape depressive outcomes. The results confirm that five major dimensions of life stress, specifically, interpersonal relationships, academic stress, punishment, loss, and health and adaptation, maintain significant positive correlations with symptom severity. These findings align with the existing literature [47, 48] and reinforce the view that environmental stressors act as triggers for depressive symptoms by engaging underlying psychological vulnerabilities.

Particularly noteworthy is the inconsistent predictive ability of punitive life events observed across diverse analytical frameworks. This discrepancy may partly reflect overlapping variance between punishment and other life‐event dimensions, particularly interpersonal relationships and academic stress, which may reduce the unique contribution of punishment in the adjusted regression model. This pattern underscores the necessity for cautious variable‐specific interpretation when navigating the clustered nature of adolescent stress exposure [49, 50].

Regarding the role of personality, the robust association identified between neuroticism and depressive symptoms lends empirical weight to the five‐factor model [51] and echoes the theoretical insights of John et al. [52]. The implementation of interpretable machine learning further clarifies this relationship by quantifying predictive importance. Specifically, neuroticism yielded the highest mean absolute SHAP value (1.681), marking it as the most influential predictor in our model and surpassing both life events and social support subscales. This predictive dominance likely arises because neuroticism represents a stable internal vulnerability rather than a transient situational response. Research suggests that adolescents characterized by high neuroticism often exhibit persistent cognitive biases, such as a selective focus on negative stimuli or a tendency toward rumination [53]. Unlike fluctuating environmental stressors, this enduring personality predisposition consistently mediates the translation of external pressure into psychological distress [54]. Furthermore, elevated neuroticism tends to impair emotional regulation and stress‐coping mechanisms, frequently leading to passive avoidance strategies that sustain depressive states [55]. In essence, while environmental stressors and limited social support may contribute to the development of depressive symptoms, neuroticism may represent a relatively stable internal vulnerability that amplifies these risks.

Finally, our data indicate that subjective support and support utilization were negatively associated with depressive symptoms, consistent with the social support theory [24]. While adequate support systems are known to alleviate psychological strain, our findings suggest that social support and neuroticism showed primarily independent protective and risk associations, respectively, with depressive symptoms. The longitudinal moderation analysis did not identify a significant interaction between neuroticism and social support, indicating that the protective association of social support was not significantly modified by neuroticism in the present sample.

### 4.3. Longitudinal Analysis of Depressive Symptoms Among College Students

Longitudinal tracking reveals a pronounced escalation in depressive symptoms among college students following the COVID‐19 outbreak, a trend that converges with multinational epidemiological evidence [56, 57]. This upward trajectory may be related to a constellation of pandemic‐associated stressors, particularly prolonged social isolation and the abrupt transition to remote learning environments. These contextual shocks likely amplified the preexisting negative impacts of academic pressure and strained support utilization on student mental health [58, 59]. These intersecting factors collectively demonstrate the degree to which the various stressors precipitated by the pandemic, specifically social disconnection and academic disruption, have exerted a sustained toll on the psychological well‐being.

Beyond general symptom elevation, our data show concomitant increases in neuroticism and academic stress, alongside a notable erosion of subjective support. The synchronous rise in academic pressure and the attenuation of support utilization may have accompanied the longitudinal increase in depressive symptoms. Unlike routine academic settings, emergency remote learning during the pandemic disrupted established institutional support structures and increased self‐regulatory demands, which may have intensified academic stress. Meanwhile, restricted social contact may have hindered in‐person peer support and help‐seeking behaviors, thereby reducing opportunities to utilize available support [59, 60]. These findings resonate with early‐pandemic observations by Zhang et al. [61] and find robust theoretical grounding in the stress‐vulnerability model [62]. Within this framework, the pandemic acts as a chronic environmental stressor that activates inherent negative cognitive biases and emotional dysregulation in individuals with high neuroticism. Increased academic and interpersonal pressures further fuel rumination, while diminished social support erodes essential coping buffers, collectively driving the longitudinal symptomatic progression.

Two key findings from the cross‐lagged model consolidate this theoretical interpretation. The analysis confirmed the longitudinal predictive role of neuroticism in symptom progression, supporting its status as a stable internal vulnerability trait. Furthermore, the results offer novel empirical support for applying this framework to public health emergencies. Chronic pandemic‐related stress may interact with neurotic vulnerability and declining social support in ways that contribute to the persistence of depressive symptoms [62].

Interestingly, the cross‐lagged model showed that subjective support was significantly negatively associated with subsequent depressive symptoms (*β* = −0.082, *p* = 0.024). This result supports the core tenets of Cohen and Wills’ stress‐buffering theory [24], which emphasizes that the subjective perception of being supported is more critical than the mere presence of objective resources. As a stable cognitive appraisal of external emotional acceptance, subjective support may help mitigate depressive symptoms and may theoretically attenuate the activation of neurotic vulnerability under pandemic‐related stressors. Such findings reinforce the superior protective value of subjective perceptions over objective support [63]. Therefore, enhancing subjective support should be a prioritized intervention target for disrupting the stress‐vulnerability cycle among college students during public health crises.

A longitudinal moderation analysis was further conducted to examine the interaction between neuroticism and social support on depressive symptoms. Consistent with prior longitudinal research [64, 65], neuroticism and social support acted as independent risk and protective factors for college students’ depressive symptoms, with no significant buffering interaction being identified. This nonsignificant finding may stem from the ceiling effect of social support in our sample, which limited interindividual variance [66]. Moreover, the stress‐buffering effect of social support is more prominent under acute stress, whereas our study focused on the chronic progression of depressive symptoms in a stable post‐pandemic context [67, 68]. Reliance on an aggregate social support index potentially obscures the discrete moderating contributions of individual subconstructs, most notably the unique role of subjective support [69].

## 5. Conclusion

Overall, the study found that several demographic and background characteristics, including family economic status, childhood experiences, parental relationships, parent–child relationships, and histories of psychiatric illness and suicidal behavior, were significantly associated with depressive symptoms among college students. Additionally, adolescent life events and neuroticism were positively associated with depressive symptoms, whereas subjective support and support utilization were negatively associated with depressive symptoms. The follow‐up study revealed a notable increase in depressive symptom scores and detection rates among college students during the pandemic period, suggesting a deterioration in mental health over the follow‐up interval. Furthermore, the cross‐lagged analysis identified neuroticism as a significant positive longitudinal predictor of depressive symptoms and subjective support as a significant negative longitudinal predictor. In contrast, the individual negative life‐event dimensions did not show significant longitudinal predictive effects.

The robust predictive utility of neuroticism, negative life events, subjective social support, adverse childhood experiences, and personal psychiatric history suggests that these core factors could be integrated into a streamlined screening instrument for incoming university freshmen. Compared with more extensive traditional assessments, such a streamlined approach may reduce the respondent burden and improve the feasibility of large‐scale screening in university settings. With further validation, model‐derived risk thresholds could potentially be used to categorize students into different risk strata and support a more efficient tiered management framework in university mental health services. This systematic approach offers counselors a standardized protocol for identifying early depressive indicators during routine interviews, thereby enhancing the precision of preventive interventions. While this tool facilitates rapid preliminary identification, its application remains strictly confined to risk stratification and should not be considered a substitute for comprehensive clinical diagnosis [70, 71].

Future research should prioritize the collection of more comprehensive datasets to optimize the provision of mental health services and improve interventions for student depressive symptoms. Specifically, empirical research should focus on validating the practical applicability of these screening frameworks in real‐world clinical or campus settings.

## 6. Limitations and Prospects

Despite the contributions of this research in identifying factors associated with depressive symptoms among college students, certain methodological limitations inherent in the study design require critical recognition. First, future research should incorporate samples from universities across multiple regions and types, thereby enhancing the generalisability of the research findings. Second, future research should increase the number of time points at which data is collected in order to gain a more comprehensive understanding of the process of change in depressive symptoms. Finally, future studies should also be conducted during noncrisis periods in order to explore the effects and mechanisms of factors influencing depressive symptoms in normative settings.

To enhance the efficacy of campus mental health services and efficaciously mitigate depressive symptoms among college students, it is advised that a mental health monitoring and counseling platform be incorporated into subsequent research and practice. The platform should be equipped with real‐time counseling and monitoring functions and provide students with a privacy‐protected and user‐friendly self‐service system to facilitate self‐management and enhance students’ mental health. In particular, for students with a history of suicidal ideation, mental illness, strained family relationships, or unfortunate childhood experiences, educational institutions should implement a comprehensive mental health profile system. Optimizing institutional support requires the synthesis of periodic psychological evaluations with multifaceted indicators such as academic trajectories and social integration to facilitate a granular assessment of student well‐being. This multidimensional approach enables the precise delivery of tailored intervention strategies for vulnerable cohorts. Given the strong longitudinal association between neuroticism and depressive symptoms, particularly when exacerbated by negative life stressors, universities should prioritize targeted resilience‐building initiatives. Complementing these systemic efforts with specialized seminars focused on interpersonal dynamics would equip students with the necessary toolkits to manage emergent psychosocial challenges effectively.

## Author Contributions

**Jing Jie:** conceptualization, methodology, software, validation, investigation, data curation, supervision, project administration, funding acquisition. **Ming Yin:** supervision, project administration, funding acquisition, revision. **Jiayue Zeng:** methodology, software, formal analysis, resources and data curation, writing, visualization. **Songlin Yang:** methodology, software, investigation, writing, visualization.

## Funding

This work was supported by the National Natural Science Foundation of China (Grant Nos. 32460207 and 32200892), the Hainan Provincial Natural Science Foundation of China (Grant No. 826MS0126), the STI 2030‐Major Projects (Grant No. 2022ZD0208602), and the Hainan Provincial Key Research and Development Program (Grant No. ZDYF2025SHFZ023).

## Disclosure

The AI‐assisted text was subsequently reviewed and verified in full by the research team to ensure accuracy and adherence to academic standards. The authors assume full responsibility for the integrity and originality of the presented content.

## Ethics Statement

The study was approved by the Ethics Committee of Haikou People’s Hospital in China on 15 March 2022 (Reference Number: SC20220089).

## Conflicts of Interest

The authors declare no conflicts of interest.

## Supporting Information

Additional supporting information can be found online in the Supporting Information section.

## Supporting information


**Supporting Information 1** Appendix Table S1. Presents the distribution of depressive symptom severity and detection rates across demographic and background characteristics of college students.

**Supporting Information 2** Appendix Table S2. Reports the pretest and posttest Cronbach’s *α* coefficients for the five dimensions of the Adolescent Self‐Rating Life Events Checklist (ASLEC).

**Supporting Information 3** Appendix Table S3. Compares baseline demographic characteristics and depressive symptom scores between follow‐up completers and nonrespondents in the youngest undergraduate cohort.

**Supporting Information 4** Appendix Table S4. Summarizes the results of longitudinal measurement invariance analyses across study scales, including changes in the comparative fit index (ΔCFI) and invariance conclusions.

**Supporting Information 5** Appendix Figure S1: Displays the residual histogram of the ordinary least squares (OLS) multiple linear regression model.

**Supporting Information 6** Appendix Figure S2: Presents the *Q*–*Q* plot of residuals from the OLS multiple linear regression model to assess deviations from normality.

## Data Availability

The datasets generated or analyzed during the current study are available from the corresponding author upon reasonable request.
