# Supplementary material for: Predicting Depressive Symptoms From Cross‐Sectional and Longitudinal Data Using XGBoost–SHAP and Cross‐Lagged Model
Source: Depress Anxiety. 2026 Oct 27;2026:4401725. doi: 10.1155/da/4401725 (PMC13711493; doi:10.1155/da/4401725)
Supplement: Supplementary file 1 [file DA-2026-4401725-s002.pdf]

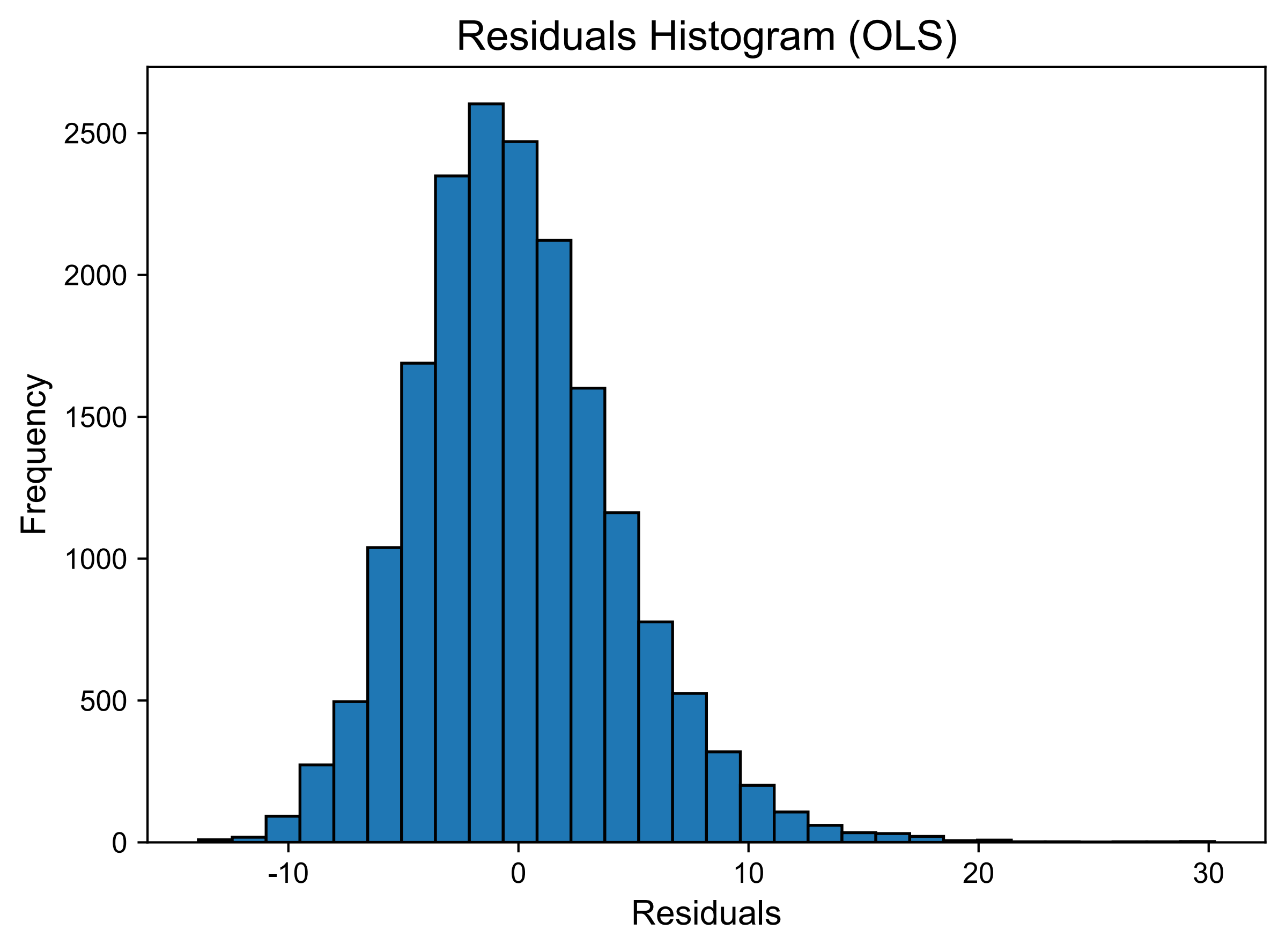
Residuals Histogram (OLS)
Frequency
2500
2000
1500
1000
500
0
-10
0
10
20
30
Residuals
