# Supplementary material for: Predicting Depressive Symptoms From Cross‐Sectional and Longitudinal Data Using XGBoost–SHAP and Cross‐Lagged Model
Source: Depress Anxiety. 2026 Oct 27;2026:4401725. doi: 10.1155/da/4401725 (PMC13711493; doi:10.1155/da/4401725)
Supplement: Supplementary file 2 [file DA-2026-4401725-s005.pdf]

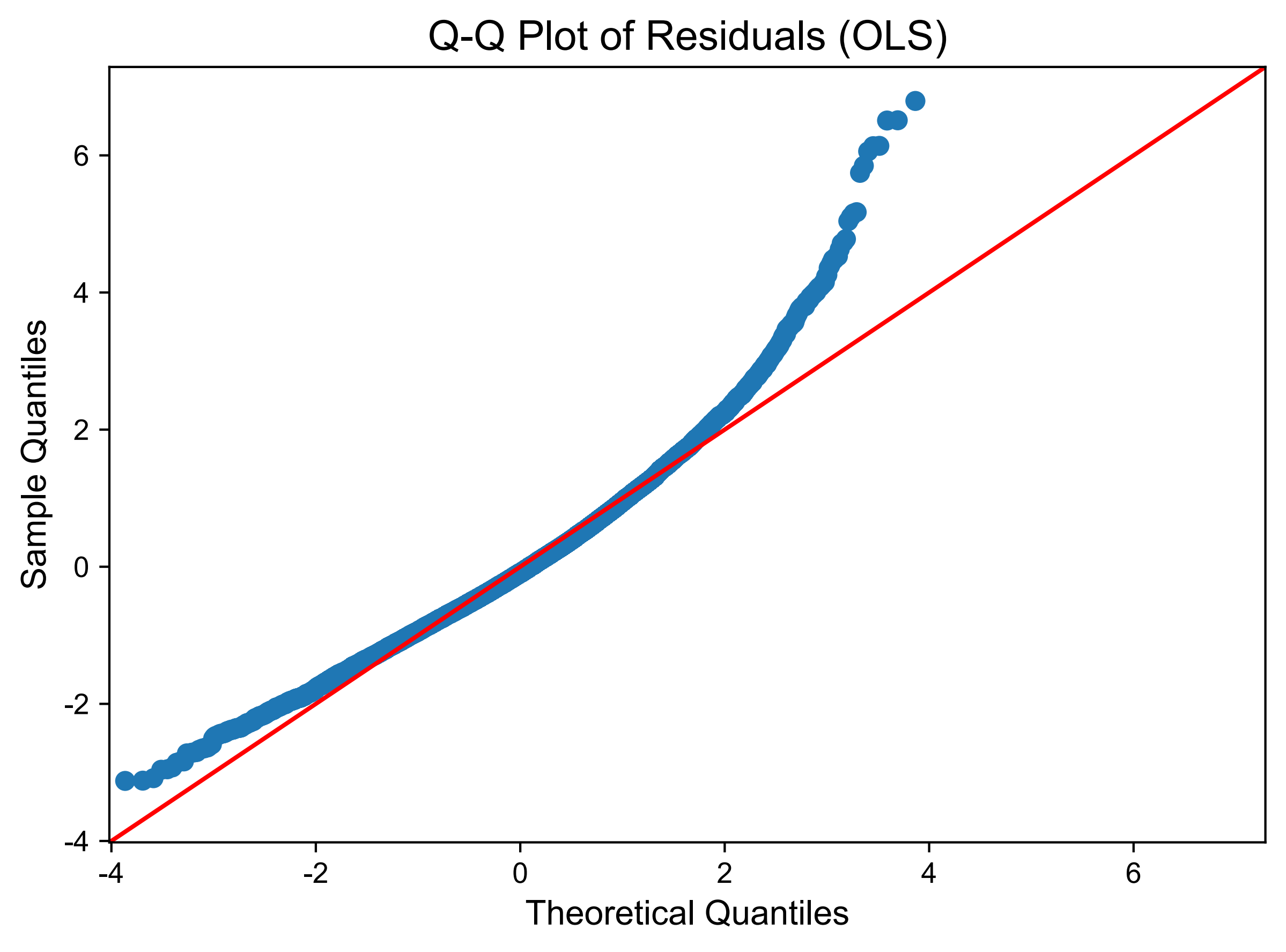
Q-Q Plot of Residuals (OLS)
Sample Quantiles
Theoretical Quantiles
6
4
2
0
-2
-4
-4
-2
0
2
4
6
